# Fault Diagnosis of a Wind Turbine Gearbox Based on Improved Variational Mode Algorithm and Information Entropy

**DOI:** 10.3390/e23070794

**Published:** 2021-06-23

**Authors:** Fan Zhang, Wenlei Sun, Hongwei Wang, Tiantian Xu

**Affiliations:** School of Mechanical Engineering, Xinjiang University, Urumqi 830047, China; zhangfan@stu.xju.edu.cn (F.Z.); wanghongwei@stu.xju.edu.cn (H.W.); xutiantian@stu.xju.edu.cn (T.X.)

**Keywords:** wind turbine gearbox, variational mode decomposition, time-shifting multi-scale sample entropy, sparrow search algorithm, support vector machine, fault diagnosis

## Abstract

The working environment of wind turbine gearboxes is complex, complicating the effective monitoring of their running state. In this paper, a new gearbox fault diagnosis method based on improved variational mode decomposition (IVMD), combined with time-shift multi-scale sample entropy (TSMSE) and a sparrow search algorithm-based support vector machine (SSA-SVM), is proposed. Firstly, a novel algorithm, IVMD, is presented for solving the problem where VMD parameters (*K* and *α*) need to be selected in advance, which mainly contains two steps: the maximum kurtosis index is employed to preliminarily determine a series of local optimal decomposition parameters (*K* and *α*), then from the local parameters, the global optimum parameters are selected based on the minimum energy loss coefficient (ELC). After decomposition by IVMD, the raw signal is divided into *K* intrinsic mode functions (IMFs), the optimal IMF(s) with abundant fault information is (are) chosen based on the minimum envelopment entropy criterion. Secondly, the time-shift technique is introduced to information entropy, the time-shift multi-scale sample entropy algorithm is applied for the analysis of the complexity of the chosen optimal IMF and extract fault feature vectors. Finally, the sparrow search algorithm, which takes the classification error rate of SVM as the fitness function, is used to adaptively optimize the SVM parameters. Next, the extracted TSMSEs are input into the SSA-SVM model as the feature vector to identify the gear signal types under different conditions. The simulation and experimental results confirm that the proposed method is feasible and superior in gearbox fault diagnosis when compared with other methods.

## 1. Introduction

In recent decades, the installed scale and grid-connected capacity of wind turbines have increased significantly. As of the end of 2019, the installed capacity of wind turbines in the world exceeded 690 million kW, and more than 100,000 wind turbines had been built [1]. Behind such a huge installed capacity, the daily maintenance of wind turbines is essential. The efficient and accurate fault diagnosis method not only can real-time monitor the operation of the wind turbines, and also can detect the potential fault of wind turbines. Wind turbines’ fault diagnosis methods mainly include the method based on the model and the method based on data [2]. Casau et al. [3] used fault detection and isolation (FDI) and fault-tolerant control (FTC) methods based on the model falsification technique using set-valued observers (SVOs) to ensure the safe operation of wind turbines. Odgaard et al. [4] proposed a standard wind turbine benchmark model, which was used to detect the running state of the wind turbines. Badihi et al. [5] used fuzzy mathematical language to model wind turbines and proposed an integrated fault detection and diagnosis (FDD) and FTC scheme. Apart from providing fault tolerance of wind turbine sensors, the scheme also greatly improves the overall performance of wind turbines under fault-free and fault-free conditions. The above methods have achieved certain results, but with the continuous development of rotating equipment, the mechanical composition is increasingly complex, therefore, it is hard to build a model which can comprehensively reflect the mechanical equipment. With the wide application of big data, data mining technology has been maturing. As a result, the data-driven fault diagnosis method of rotating equipment has been favored by many researchers. The time-frequency characteristics of vibration signals in different states have clear differences, additionally, the vibration signals are easy to be collected. So, the fault diagnosis method of rotating mechanical equipment based on vibration signal analysis is the most widely used method at present.

The gearbox is a critical component in the drive system of a wind turbine; due to the influence of its working environment, local faults such as cracks or spalling frequently occur. According to research, gearbox faults account for one-quarter of the total wind turbine faults, and the operation and maintenance costs account for about 25%–35% of the total cost of wind turbines. The downtime caused by gearbox faults is longer than that caused by other faults [6,7]. Therefore, it is essential to effectively monitor the running condition of the gearbox.

The working environment of a wind turbine gearbox is complex, which is often exposed to heavy loads, high temperatures, and speed changes. Additionally, due to the influence of load fluctuations and sliding friction between teeth, the collected gearbox vibration signal is non-stationary and nonlinear. Consequently, researchers have proposed various methods for the analysis of non-stationary and nonlinear signals. Ridha et al. [8] used empirical mode decomposition (EMD) combined with a shock detector (SD) to detect impact damage to bevel gearboxes in the non-static state. Amrinder et al. [9] presented ensemble empirical mode decomposition (EEMD) to initially decompose vibration signals of rolling bearings and combined it with fuzzy entropy to predict and analyze the severity of faults in the inner and outer rings of rolling bearings. Yao et al. [10] proposed a complementary fully integrated empirical mode decomposition based on adaptive noise (CCEEMDAN) and applied it to the fault diagnosis of rolling bearings. These researches have achieved certain results, however, EMD is a signal analysis method based on a recursive algorithm. Due to the limitation of its principle, signals decomposed by EMD and some of its derivative algorithms may induce several problems such as mode mixing and end effects. The variational mode decomposition (VMD) algorithm proposed by Dragomiretskiy et al. [11] can effectively overcome the above problems. Additionally, the algorithm outperforms EMD in terms of noise robustness; therefore, it has been widely used in signal decomposition. Zhang et al. [12] used VMD combined with sample entropy to extract the features of weak faults of planetary gearboxes. Pan et al. [13] proposed the combination of VMD and wavelet packet for gear fault diagnosis. VMD has merits compared with other algorithms in signal decomposition, whereas its signal decomposition performance strongly depends on the selection of mode decomposition parameter *K* and the mode frequency bandwidth control parameter *α* of the VMD algorithm. When *α* is small, and the bandwidth of the Wiener filter is large, which can better detect the impact components in the signal; however, simultaneously, the decomposed modes are more likely to contain noise components [14]. When *K* is small, the number of decomposed mode components is small, which may cause partially missing information. When the value of K is large, it may induce problems such as over-decomposition and frequency mixing. In previous research work [15,16], most scholars used the default VMD algorithm parameters (K, α) to decompose the signal according to their own professional knowledge or previous experience. Due to the different signal characteristics of different objects, decomposing the signal using default parameters cannot achieve adaptive decomposition according to the characteristics of the signal, which significantly reduces the efficiency and precision of VMD algorithm signal decomposition. With the rapid development of meta-heuristics, many scholars have selected the VMD parameters automatically using the population intelligence algorithm to achieve the best VMD signal decomposition effect. Anil et al. [17] applied a genetic algorithm (GA) to achieve the adaptive optimization of VMD decomposition parameters. Yan et al. [18] presented the cuckoo search algorithm (CSA) to determine the decomposition parameters of VMD, yet the search effect of the population intelligent search algorithm heavily relies on the preset parameters of the optimization algorithm and the fitness function selection. Accordingly, in this paper, an improved variational mode decomposition (IVMD) algorithm is proposed, which combines the kurtosis index with the energy loss coefficient (ELC) to determine the VMD parameters.

With the rapid development of nonlinear dynamics, nonlinear dynamic analysis methods have been increasingly applied to the fault diagnosis of mechanical equipment to extract fault features, for instance, entropy [19]. Approximate entropy (AE) was the entropy method first introduced in the field of mechanical fault diagnosis [20], followed by permutation entropy (PE), sample entropy (SE), fuzzy entropy (FE), discrete entropy (DE), and their derived algorithms [21,22,23,24,25,26]. Among these entropies, SE has been widely used in feature extraction due to its simple calculation and low time cost. The original sample entropy can only analyze a time series on a single scale, which leads to missing information to some extent. Multi-scale sample entropy (MSE) uses coarse graining based on the mean value to reconstruct the signal on the basis of SE, which enables the analysis of signals on different scales. MSE can be thought of as down-sampling processing; according to sampling theory, the sampling frequency is reduced and the bandwidth of the signal narrows, which leads to MSE ignoring the high-frequency information of the original signal. In addition, with the increase in the scale factor, the length of coarse-grained time series shortens; therefore, it is difficult to comprehensively obtain the information of the original signal. Considering the above issues, we propose the entropy method termed TSMSE, which combines time-shift technology and sample entropy to extract the features of gearbox signals to enable more accurate analysis and recognition of the running state of a gearbox.

At present, a mainstream fault diagnosis method involves inputting the extracted feature vector into the classifier to recognize the fault type. Xu et al. [27] presented a new method of intelligent fault diagnosis called deep convolutional nearest neighbor matching network (DC-NNMN) based on few-shot learning and used that method for the fault diagnosis of a gear with a small labeled sample. Yu et al. [28] proposed wavelet transform combined with an extreme learning machine (ELM) for automatic fault diagnosis of wind turbines. Liang et al. [29] presented the combination of convolutional neural network (CNN) and multi-label classification (ML) for the efficient compound fault diagnosis of a gearbox. Sheng et al. [30] obtained the features vectors of a bearing vibration signal by VMD-phase space reconstruction (PSR)-singular value decomposition (SVD) and used the improved k-nearest neighbor algorithm for the fault diagnosis of rolling bearings. Among numerous classifiers, support vector machine (SVM) has been widely used in fault diagnosis as a result of its strong generalization ability on nonlinear problems and its excellent reliability given small sample data. The SVM fault-recognition effect is heavily dependent on penalty factor *c* and kernel parameter *σ*; therefore, some SVM optimization methods were proposed to resolve this issue. Yan et al. [31], Li et al. [32], and Luo et al. [33] used particle swarm optimization (PSO), the cuckoo search algorithm (CSA), and gray wolf algorithm (GWA), respectively, to optimize the crucial SVM parameters; however, the above algorithms are prone to problems such as slow convergence, local optimal solutions, etc. The sparrow search algorithm (SSA) is a population intelligence algorithm proposed in 2020. Studies showed that the SSA is superior to the existing algorithms in terms of search accuracy, convergence speed, and stability [34]. Therefore, we chose to use the sparrow search algorithm to adaptively optimize the key support vector machine parameters (SSA-SVM).

To summarize, in this paper, we propose a neoteric fault diagnosis method for wind turbine gearboxes. The method contains three stages in general. First, the kurtosis index and energy loss coefficient (ELC) are used to select VMD parameters *K* and *α,* and the signal is decomposed by VMD with the determined parameters. The best IMF following the minimum envelope entropy criterion is chosen. Secondly, time-shift multi-scale sample entropy (TSMSE) is used to extract features of the optimal IMF. Ultimately, the sparrow search algorithm is applied to optimize the support vector machine parameters (SSA-SVM), then the extracted feature entropy vector is input into the optimized SVM model to analyze and identify the gearbox fault types.

The rest of this paper is organized as follows: Section 2 introduces the basic principle of the VMD algorithm and the process of the IVMD algorithm. Section 3 briefly reviews the MSE and TSMSE algorithm implementation process. Section 4 describes the sparrow search algorithm and the process of the sparrow search algorithm-based support vector machine. Section 5 outlines the fault diagnosis model of a wind turbine gearbox proposed in this paper. Section 6 describes the experiment used to verify the feasibility and effectiveness of the model proposed in this paper. Section 7 provides the conclusion of this study.

## 2. VMD and IVMD Algorithms

### 2.1. VMD Algorithm

Vibration signals of wind turbine gearboxes are characterized by strong randomness, poor stability, and aliasing of physical information. Therefore, signal decomposing should be preliminarily carried out to extract the signal features. As stated in the Introduction, EMD and other signal decomposition methods experience problems such as the end effect and mode aliasing. The VMD algorithm is a new, adaptive, non-recursive, signal-decomposition method that analyzes and decomposes the signal in the time-frequency domain using the classical Wiener filter, Hilbert transform, and frequency mixing principle. The VMD algorithm decomposes a real signal into several intrinsic mode functions (IMFs) with certain center frequencies and limited bandwidth through iteration; these modes are independent of each other and have sparseness. Therefore, VMD can effectively preprocess nonlinear signals. By estimating the bandwidth of each mode, the variational constraint equation model, as expressed in Formula (1), can be solved, which minimizes the sum of the modes’ bandwidths under the constraint that the sum of all modes is the original signal.

(1) $$\begin{array}{l} \min_{\left\{u_{k}\right\},\left\{\omega_{k}\right\}}\left\{{\sum_{k=1}^{K}\left\|\partial_{t}\left[\left(\delta (t)+\frac{j}{\pi t}\right)u_{k}(t)\right]e^{-j\omega_{k}t}\right\|}_{2}^{2}\right\}\\ s.t.\sum_{k=1}^{K}u_{k}(t)=f(t) \end{array}$$

Here, {*u_k_*} denotes decomposed IMF components, {*ω_k_*} represents the center frequency of the IMF components, *∂t* is the partial derivative of time t, *δ*(*t*) represents the unit pulse function, *j* is the imaginary unit, and *f(t)* indicates the raw signal in time-domain.

The Lagrangian multiplier *λ(t)* and penalty parameter *α* are introduced to transform Formula (1) into an unconstrained variational model, expressed as:

(2) $$\begin{array}{r} L\left(\left\{u_{k}\right\},\left\{\omega_{k}\right\},\lambda (t)\right)=\alpha {\sum_{k=1}^{K}\left\|\partial_{t}\left[\left(\delta (t)+\frac{j}{\pi t}\right)u_{k}(t)\right]e^{-j\omega_{k}t}\right\|}_{2}^{2}+\\ {\left\|f(t)-\sum_{k=1}^{K}u_{k}(t)\right\|}_{2}^{2}+\langle \lambda (t),f(t)-\sum_{k=1}^{K}u_{k}(t)\rangle \end{array}$$

The alternative direction method of the multiplication operator is applied to update *u_k_*, *ω_k_*, and *λ_k_*_,_ iteratively until the variational model obtains the optimal solution. The outputs are the mode components obtained by VMD.

### 2.2. IVMD Algorithm

**Figure 1:**
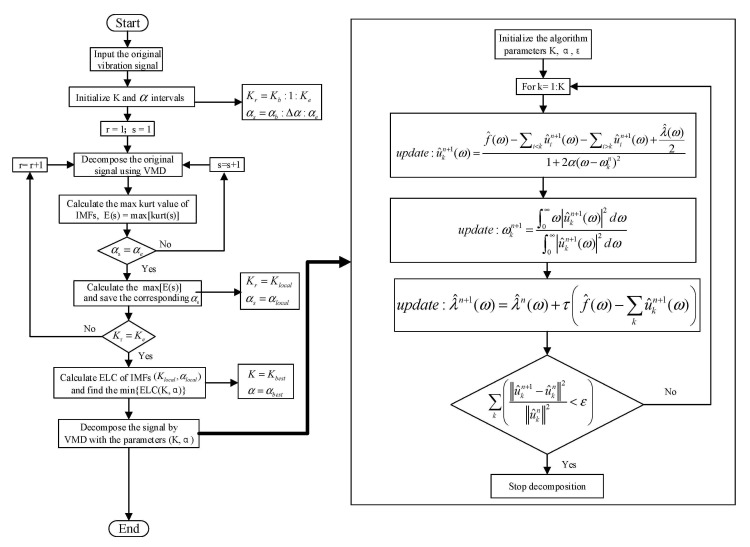
Flowchart of the IVMD algorithm.

To solve the existing problem in the VMD algorithm, we propose a method that applies the kurtosis index combined with the energy loss coefficient (ELC) to adaptively select the decomposition parameters (*K, α*) of the VMD algorithm. The flow chart is depicted in Figure 1.

The kurtosis index reflects the impact characteristics of the signal and is sensitive to weak faults in the early stage. Consequently, a series of local optimal parameter pairs are preliminarily selected according to the maximum kurtosis criterion. The calculation formula of kurtosis is as follows:

(3) $$e(i)=\frac{1}{N}{\sum_{i=1}^{N}\left(\frac{u_{i}-\bar{u}}{\sigma }\right)}^{4}$$

where *u_i_* denotes the mode component *IMF_i_*, *N* is the mode length, $\bar{u}$ represents the mean of *u_i_*, and *σ* is the standard deviation of *u_i_*.

For the search range and step size of the VMD decomposition parameter, *K_r_*∈[*K_b_*, *K_e_*], the search step size is 1, *α_s_*∈[*α_b_, α_e_*], and the search step size is set to Δ*α*. Suppose the mode decomposition number is *k*, the bandwidth control parameter is *α_s_*, and the signal is decomposed by VMD with this parameter pair. Then, the kurtosis value of each IMF is calculated using Formula (3) and the maximum kurtosis value is considered the kurtosis value under (*k*, *α_s_*). Then, the (*k*, *α_s+_*_Δ*α*_) parameter is used to decompose the signal using VMD, and the kurtosis value of each IMF is calculated again. The maximum kurtosis value is taken as the kurtosis value under (*k*, *α_s+_*_Δ*α*_). The cycle continues until *α_s_* = *α_e_*. The $\alpha_{\mathrm{lo}bal}^{k}$ corresponding to the maximum kurtosis value in the series of obtained kurtosis values is taken as the optimal *α* under this mode number *k*. Then, the mode number is set to *k +* 1, and the next cycle is continued until *k* = *K_e_*. Then a series of local optimal parameters can be obtained. The mathematical expression is expressed as follows:

(4) $$\begin{array}{l} \vec{e_{s}^{k}}=\max(e_{s}^{1},e_{s}^{2},\ldots ,e_{s}^{k})\\ e_{lobal}^{k}=(\vec{e_{b}^{k}},\vec{e_{b+\Delta \alpha }^{k}},\ldots ,\vec{e_{s}^{k}},\ldots ,\vec{e_{e}^{k}})\\ \alpha_{lobal}^{k}=\arg\max(e_{lobal}^{k}) \end{array}$$

After obtaining a series of local optimal parameter pairs, the signal is decomposed by the combination of local parameters. Then, the ELC of the mode component is calculated, and the parameter with the least energy loss is taken as the global optimal VMD decomposition parameter. The ELC calculation formula is expressed as:

(5) $$ELC=\frac{{\left\|f(t)-\sum_{k=1}^{K}u_{k}\right\|}_{2}^{2}}{{\left\|f(t)\right\|}_{2}^{2}}$$

where *f(t)* denotes the signal.

## 3. TSMSE Algorithm

When wind turbine gearbox faults occur, the complexity and chaos characteristics of the collected vibration signal change. Therefore, the linear signal processing method cannot be used to accurately analyze the signal, so the nonlinear dynamic analysis method is introduced to extract and analyze the nonlinear characteristics of the signal. As stated in the Introduction, sample entropy and multi-scale sample entropy are widely used nonlinear dynamic parameter that can reflect the irregularity and complexity of signals, but they experience some issues, such as sample entropy can only reflect the information of a signal on a single scale, multi-scale sample entropy cannot measure the complexity of a signal at high scale. To solve the problems faced by sample entropy and multi-scale sample entropy for extracting fault features, time-shift multi-scale sample entropy (TSMSE) is proposed. 

### 3.1. Multi-Scale Sample Entropy Algorithm

The multi-scale sample entropy (MSE) based on the sample entropy uses the coarse-graining technology to obtain a series of the coarse-graining time series, and then the sample entropy of each coarse-graining time series is calculated, the implementation process is described as follows:

For a given raw time series {*x*(*i*), i = 1,2, …, N}, the coarse-graining process follows the Formula (6).

(6) $$u_{j}^{k}=\frac{1}{k}\sum_{i=(j-1)k+1}^{jk}x_{i}$$

where *k* represents the scale factor; *j* = 1, 2, 3, …, *N*/*k*; *N* is the length of the series.

(7) $$\begin{array}{l} MSE(x,k,m,r)=SE(u^{k},m,r)\\ r=(0.2∼0.25){}^{*}std(u^{k}) \end{array}$$

where *r* is the similarity tolerances; *m* is pattern dimension; std represents the standard deviation. SE means the sample entropy value of the time series. The detailed sample entropy calculation steps are described in [22].

### 3.2. Time-Shift Multi-Scale Sample Entropy Algorithm

In the time-shift multi-scale sample entropy algorithm, raw time series {*x(i)*, *i* = 1,2, …, N} are reconstructed according to Formula (8). The sample entropy is calculated for each time shift series after reconstruction.

(8) $$u^{\beta }{}_{k}=(x_{\beta },x_{\beta +k},x_{\beta +2k},\ldots ,x_{\beta +ck})$$

where *k* denotes the scale factor; *β* = 1, 2, 3, …, *k*, *c* = $\frac{N-\beta }{k}$, which indicates the largest integer not exceeding $\left(\frac{N-\beta }{k}\right)$, where *N* is the length of the series. 

The TSMSE is expressed as:

(9) $$\begin{array}{l} TSMSE(x,m,r,k)=\frac{1}{k}\sum_{\beta =1}^{k}SE(u^{\beta }{}_{k},m,r)\\ r=(0.2∼0.25){}^{*}std(u^{\beta }{}_{k}) \end{array}$$

**Figure 2:**
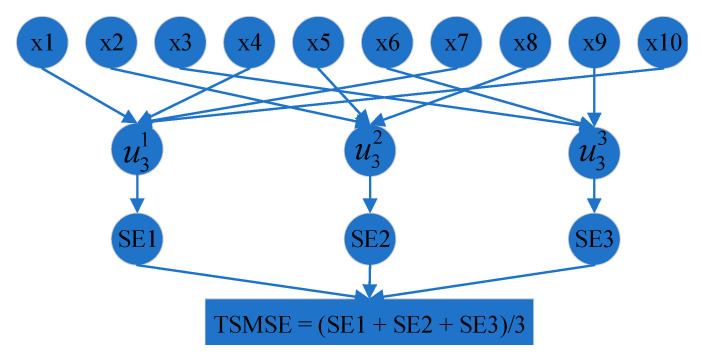
Calculation process of TSMSE.

We use an example to clearly present the process of signal reconstruction, where *N* is 10 and the scale is set to 3. Given a time series {*x(i)*, *i* = 1, 2, …, 10}, the calculation process of its TSMSE value is shown in Figure 2.

It is worth noting that when the scale factor is 1, the time series reconstructed by the MSE and TSMSE algorithms are the same as the original time series, therefore, the entropy value calculated by the MSE and TSMSE algorithms also are the same as that value calculated by the SE algorithm. 

## 4. SSA-SVM Algorithm

**Figure 3:**
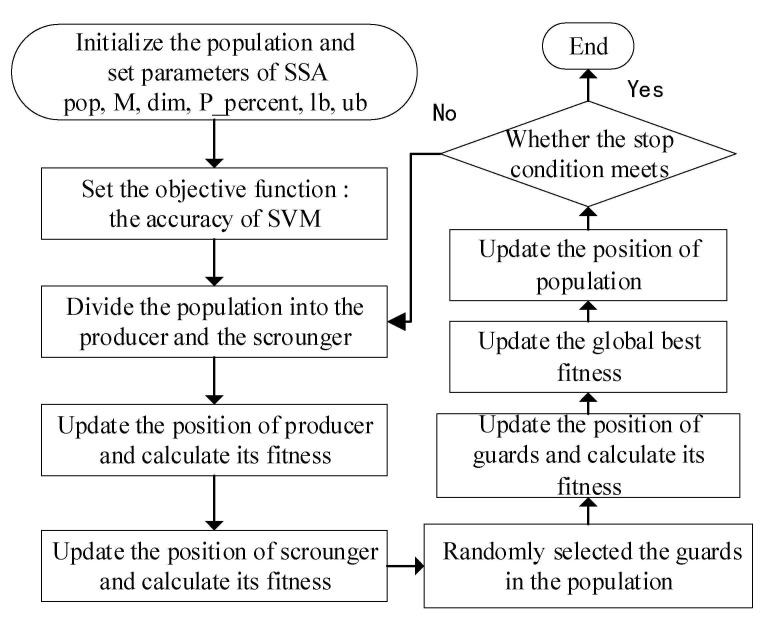
Flowchart of SSA-SVM algorithm.

The SVM algorithm maps sample space data into high-dimensional space through the kernel function and then classifies the data in high-dimensional space. As such, the nonlinear separable problem can be transformed into a linear separable problem. Therefore, the SVM algorithm performs well with nonlinear data classification problems and it is widely used in the fault diagnosis of rotating machinery equipment [35,36,37], but the problem of parameter selection must be addressed. According to the above analyses, for solving the difficulties experienced with SVM classification model application, we selected the sparrow search algorithm (SSA) to search for the optimal SVM parameters (*c*, *σ*) and set the SVM classification error rate as the fitness function; the flow chart and mathematical model are shown in Figure 3 and Formula (10), respectively.

(10) $$fit(c,\sigma )=\frac{1-accuracy}{100}$$

Here, *accuracy* denotes the SVM classification accuracy.

The SSA is inspired by the behavior of sparrow groups when foraging and when facing predation, which divides the sparrow population into producers and scroungers, while each sparrow individual position represents a solution of an SVM parameter. The producers are actively looking for food sources, while scroungers obtain food from producers. Naturally, when a predator is found by a sparrow, it will send an alarm, then the population will fly away. Through the above process, the optimal solution to a problem can be determined. The positions of the producer, scrounger, and the sparrow who identifies the danger are updated according to Formulas (11)–(13).

(11) $$x_{i,j}^{t+1}=\left\{\begin{array}{l} x_{i,j}^{t}{}^{*}\exp(\frac{-i}{\alpha {}^{*}\mathrm{it}er_{\max}}){\;\mathrm{if}\;R}_{2}<\mathrm{ST}\\ x_{i,j}^{t}+Q{}^{*}L{\;\mathrm{if}\;R}_{2}\geq \mathrm{ST} \end{array}\right.$$

Here, *t* denotes the current iteration; *j* = 1, 2, …, d. $x_{i,j}^{t}$ represents the value of the *j*th dimension of the *i*th sparrow at iteration *t*; iter_max_ is the maximum number of iterations; *α* is a random number in (0, 1]; *Q* is a random number that obeys normal distribution; *L* represents a unit matrix of 1 × d; *R_2_* indicates the value of alarm, which usually is taken from [0,1]; and *ST* (*ST*∈[0.5, 1.0]) represents the safety threshold.

(12) $$x_{i,j}^{t+1}=\left\{\begin{array}{l} Q{}^{*}\exp(\frac{x_{worst}^{t}-x_{i,j}^{t}}{i^{2}})\;\mathrm{if}\;i>\frac{n}{2}\\ x_{P}^{t+1}+\left|x_{i,j}^{t}-x_{P}^{t+1}\right|{}^{*}A^{+}{}^{*}L\;other \end{array}\right.$$

Here, *x_p_* denotes the best position of the producer, *x_worst_* represents the current global worst position, *A* is a 1 × d matrix, and the elements in the matrix are randomly assigned from [1, −1], and *N* indicates the population size. $A^{+}=A^{T}{\left(AA^{T}\right)}^{-1}$.

(13) $$x_{i,j}^{t+1}=\left\{\begin{array}{l} x_{best}^{t}+\beta {}^{*}\left|x_{i,j}^{t}-x_{best}^{t}\right|\;\mathrm{if}\;f_{i}>f_{g}\\ x_{i,j}^{t}+K{}^{*}\left(\frac{\left|x_{i,j}^{t}-x_{worst}^{t}\right|}{(f_{i}-f_{w})+\varepsilon }\right)\;\mathrm{if}\;f_{i}=f_{g}\; \end{array}\right.$$

Here, *x_best_* denotes the global best position at present; *β* is a random number, which follows a mean of 0 and a variance of 1 in the normal distribution; *K* represents a random number within [−1, 1]; *f_i_, f_g_,* and *f_w_* indicate the fitness value of the current individual, the fitness value of the global optimal solution and the fitness value of the worst solution, respectively. In addition, an arbitrary small constant *ε* is introduced to avoid zero division error [34].

## 5. The Proposed Wind Turbine Gearbox Fault Diagnosis Model 

**Figure 4:**
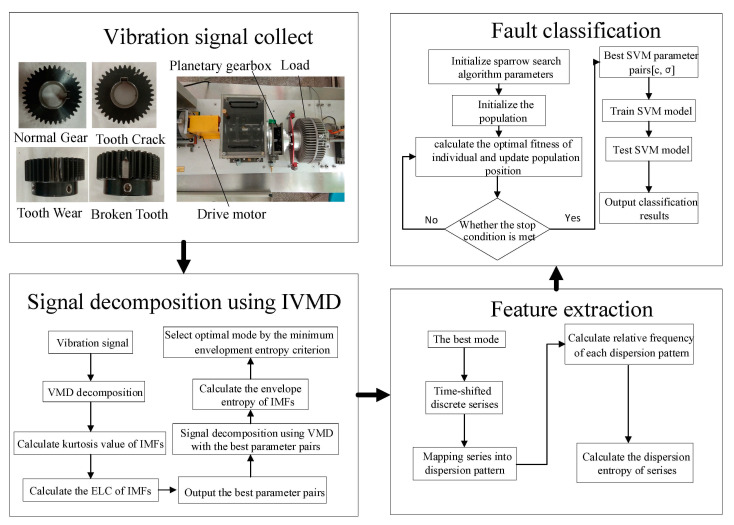
Flowchart of fault diagnosis.

Based on the above theories, we constructed a novel method for fault diagnosis of wind turbine gearboxes that combines IVMD-TSMSE with SSA-SVM. The flowchart of the proposed method is described in Figure 4 and the specific steps are as follows:

**Step 1**: The integrated wind turbine power transmission fault diagnosis platform collects the vibration signals of gears under different working conditions.
**Step 2**: The kurtosis index and energy loss coefficient are used to determine the optimal parameter pairs (*K_best_*, *α_best_*) of VMD, then the original vibration signals are decomposed into several modes.
**Step 3**: In line with the minimum envelopment entropy criterion, the optimal mode is chosen for subsequent analysis.
**Step 4**: The feature vectors that contain rich fault information are extracted from the optimal mode using the TSMSE algorithm.
**Step 5**: The best parameters’ penalty factor *c* and kernel parameter *σ* of SVM are determined by the SSA.
**Step 6**: The extracted feature vectors are randomly divided into training samples and testing samples. The training samples are used to train the optimized SVM model with the SSA, whereas the testing samples are used to test the trained SVM model for proving the effectiveness and superiority of the method proposed in this paper on the classification of different conditions of wind turbine gearboxes.

## 6. Experimental Validation of the Proposed Model

In this study, the integrated wind turbine power transmission fault diagnosis platform was adopted for data acquisition and the experimental platform is described in Figure 4. We used the three-axis acceleration sensor produced by the American PCB company to collect the gear signals, set the motor speed to 1000 rpm, the load to 0.8 hp, the sampling frequency to 20,480 Hz, and the sampling time to 4 s.

**Figure 5:**
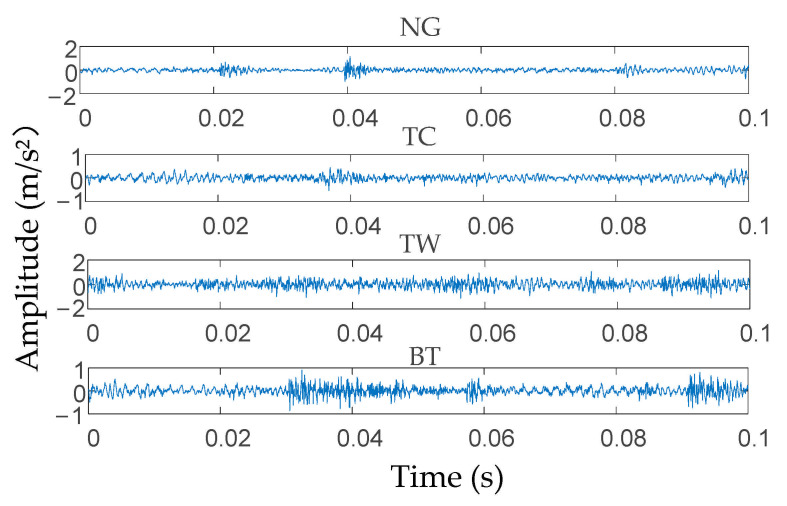
Time-domain waveform of the original signal.

The vibration signals of the input shaft of the secondary parallel shaft gearbox under four conditions (i.e., normal gear (NG), tooth crack (TC), tooth wear (TW), and broken tooth (BT)) were collected separately. Forty sets of vibration data were collected for each condition and each set included 2048 sampling points. Additionally, the ratio of training samples to testing samples was 1:1. The time-domain waveform diagram of the original vibration signals under the four conditions is shown in Figure 5. To better display the characteristics of the different signals, the waveform diagram only shows one set of data (2048 data points) for each work condition.

### 6.1. Experimental Signal Decomposition Based on the IVMD Algorithm

**Figure 6:**
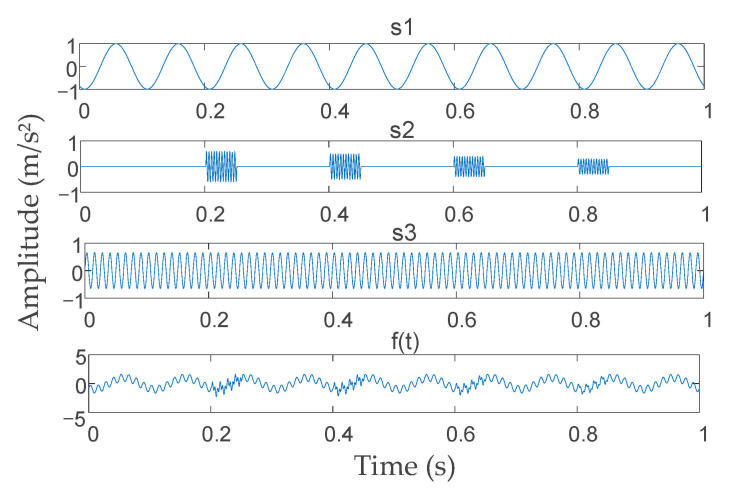
Time-domain waveform of the simulation signal.

The simulation signal obtained from [38] was used to validate the signal decomposition effectiveness of the proposed IVMD algorithm, and the simulation signal expression and its time-domain waveform are shown in Formula (14) and Figure 6.

(14) $$\begin{array}{l} s_{1}(t)=\sin(20\pi t+\frac{100}{3}\pi ),\\ s_{2}(t)=\left\{\begin{array}{l} 0.6\sin(480\pi t),\;t\in [0.2,0.25],\\ 0.5\sin(480\pi t),\;t\in [0.4,0.45],\\ 0.4\sin(480\pi t),\;t\in [0.6,0.65],\\ 0.3\sin(480\pi t),\;t\in [0.8,0.85], \end{array}\right.\\ s_{3}(t)=0.65\sin(160\pi t),\\ f(t)=s_{1}+s_{2}+s_{3} \end{array}$$

**Figure 7:**
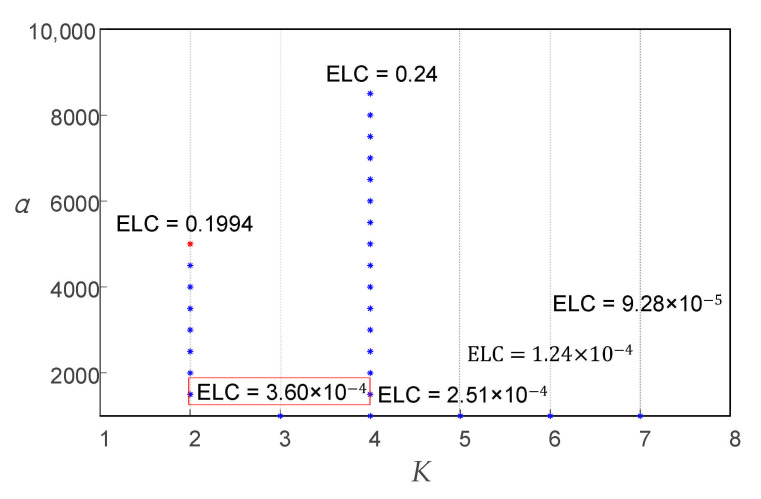
IVMD parameter optimization process.

The search range and step of *K* and *α* were preliminarily set to *K* ∈ [2, 7] and *α* ∈ [1000, 10,000], respectively, with a search step Δ*α* = 500. Selecting the best VMD parameters according to the IVMD algorithm process described above. The parameter optimization process of the IVMD algorithm for the simulation signal is shown in Figure 7. 

**Figure 8:**
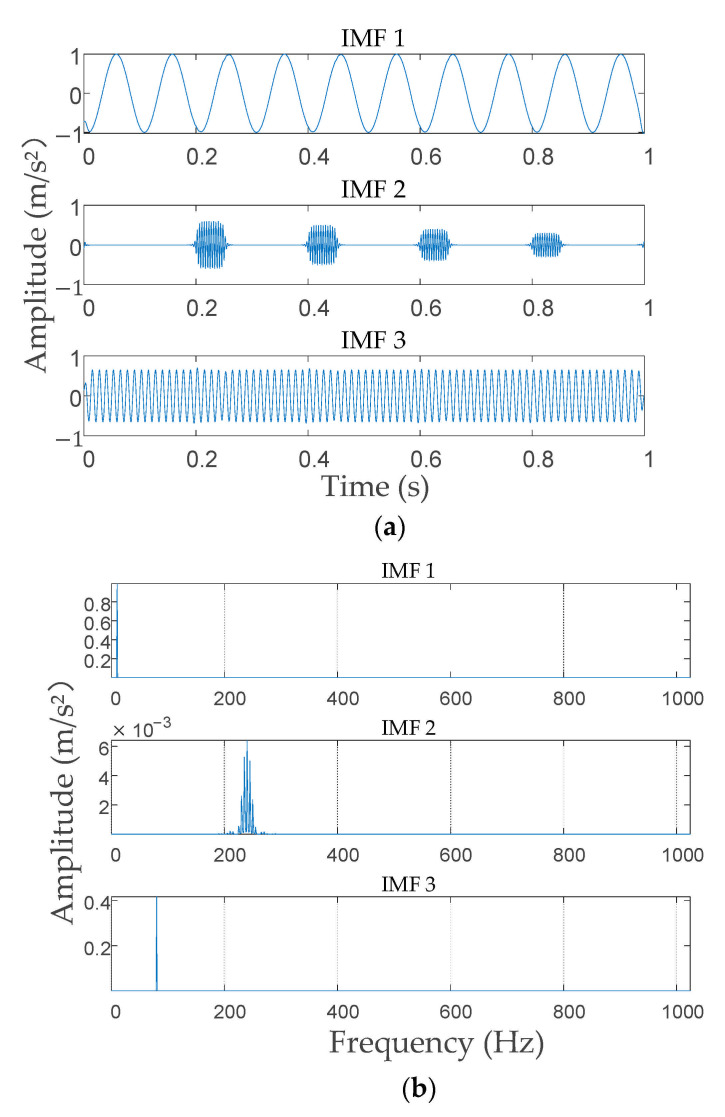
Decomposition results of simulation signal. (**a**) Decomposition result in the time domain; (**b**) decomposition result in the frequency domain.

From Figure 7, under the local optimal parameter combinations, with the increase in *K*, the ELC value decreases at the beginning and then increases, and then decreases continually. According to the trend in Figure 7, it can be predicted that when *K* continues to increase, the ELC will also decrease. Therefore, after the mode decomposition number *K* was 5 (including 5), the simulation signal showed over-decomposition and mode mixing. Based on the result (3, 1000), which is marked in the red box and selected as the optimal VMD decomposition parameter of the simulation signal, the signal was decomposed by VMD with that parameter. The decomposition results are presented in Figure 8. Figure 8a shows that after the simulation signal was decomposed with the parameter (3, 1000), each mode component signal obtained corresponded to one component of the original signal and there was no over or under-decomposition, which indicated that the simulation signal was completely decomposed in the time domain. Figure 8b shows that the simulation signal from low frequency to high frequency was clearly divided by IVMD and the mode components clearly reflected the original signal in the frequency spectrum of each component of the center frequency. Additionally, the modes showed clear differences in the frequency domain, and no mode mixing or false mode occurred, which illustrated the simulation signal in the frequency domain to obtain the complete decomposition. As a result, the simulation signal proved that the proposed IVMD method can effectively determine the decomposition parameters (*K*, *α*) of VMD. 

**Figure 9:**
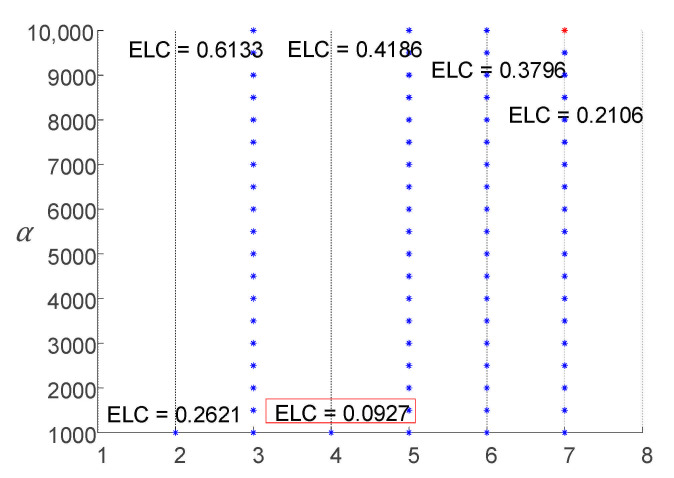
Parameter optimization process of IVMD.

**Figure 10:**
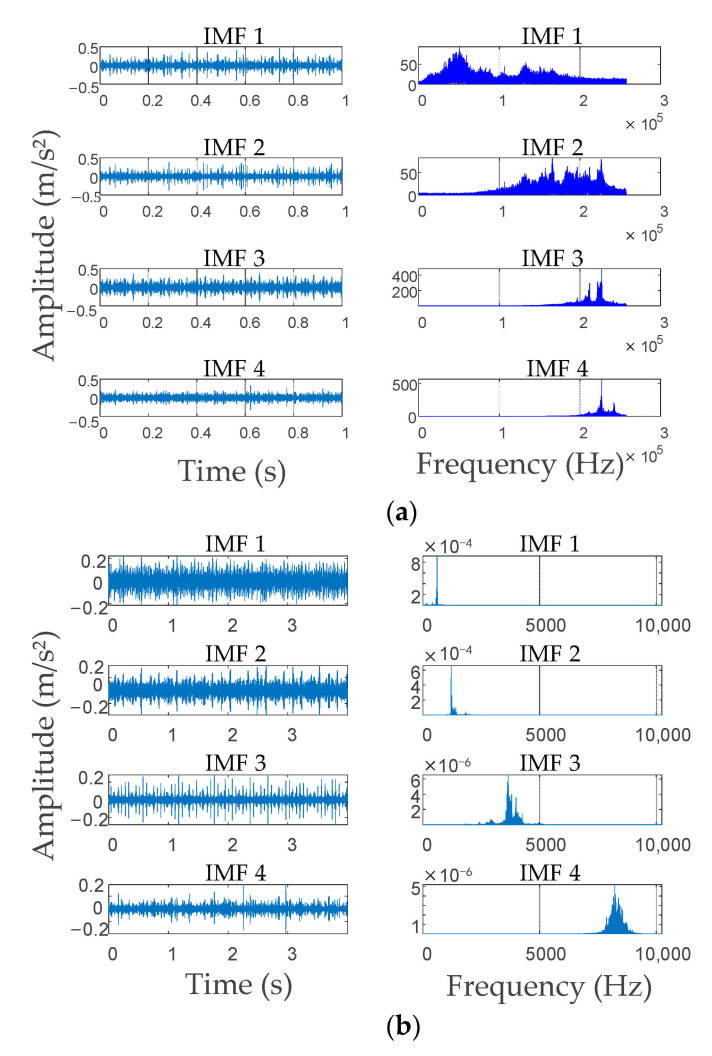

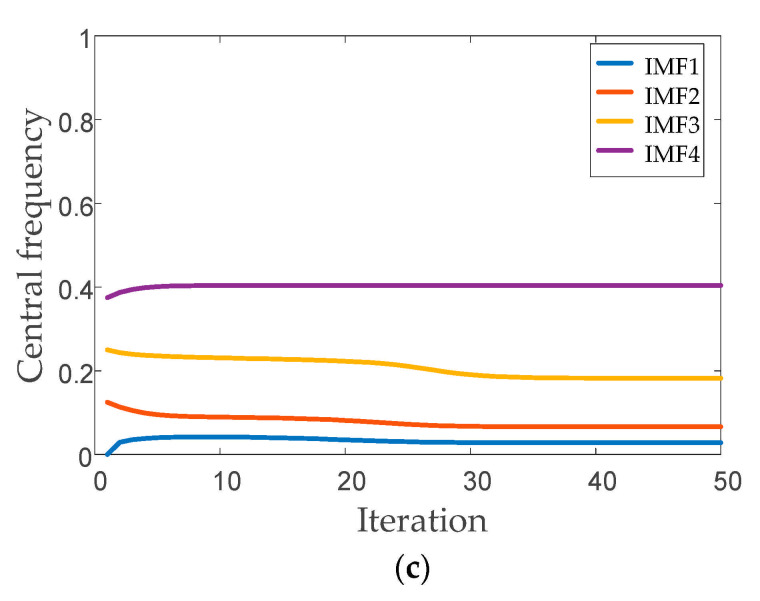
Decomposition results of a tooth crack fault. (**a**) First four decomposition results of EMD; (**b**) decomposition results of IVMD; (**c**) the iteration curve of the central frequencies.

In accordance with the above simulation signal analysis results, we used the IVMD algorithm to decompose gear signals under four conditions. Taking the tooth crack fault as an example, the optimization process of IVMD decomposition parameters is shown in Figure 9, where (4, 1000) marked in the red box is taken as the optimal VMD decomposition parameter. The EMD and IVMD algorithms were used to decompose signals with a tooth crack fault, separately, to validate that the proposed IVMD algorithm provides benefits in signal decomposition; the two methods’ decomposition results are shown in Figure 10. EMD decomposed the signal into 17 groups of IMFs; due to space limitations, only the first four IMFs are expressed. From Figure 10a, between IMF1 and IMF2, there is an obvious mode mixing in the intermediate frequency stage. The same situation also occurs between IMF3 and IMF4. In addition, there are no obvious differences between the different components in the frequency domain, which proves that EMD cannot clearly decompose the signal from low frequency to high frequency. Figure 10b shows that all the decomposed mode components by IVMD. Each IMF shows good physical significance and the information overlapping in the full frequency band was decomposed in different frequency bands by IVMD. Additionally, Figure 10c shows the iteration curve of the central frequencies of each IMF during the IVMD decomposition; there is no crossing of curves and each curve moves away from each other, which means that no mode mixing occurred in the process of IVMD decomposition. The effectiveness and advantages of the proposed IVMD algorithm in signal decomposition are verified through the above analysis.

**Figure 11:**
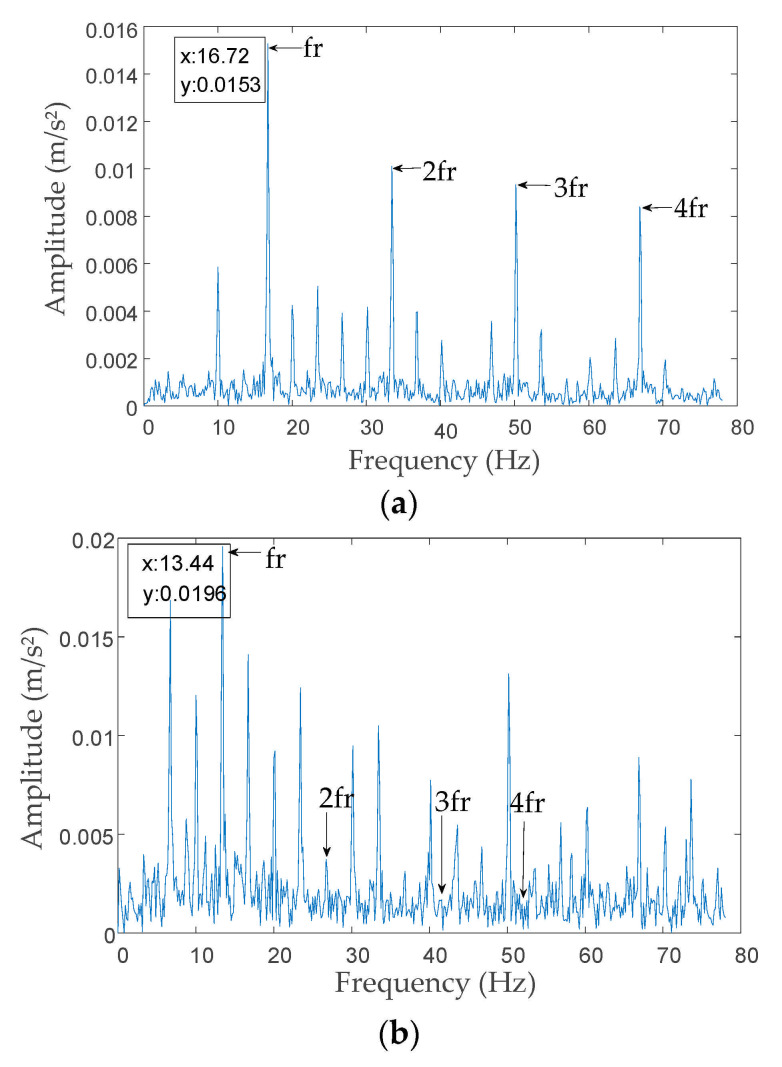
Envelope analysis results. (**a**) Envelope analysis results by IVMD; (**b**) envelope analysis results by EMD.

To further verify the above findings, IMF3 was selected as the optimal IMF decomposed by IVMD and EMD according to the minimum envelope entropy criterion to perform envelope analysis, and the results are shown in Figure 11. From Figure 11a, the mode component fault characteristic frequency and the harmonics (2fr, 3fr, and 4fr) are clearly observed via IVMD decomposition. The results basically remove the noise interference compared with Figure 11b. Conversely, from Figure 11b, the mode component decomposed by EMD shows the mode-mixing problem. The signal fault characteristic frequency and harmonics were submerged in noise, which resulted in difficulties in extracting the effective fault features. In summary, the above analysis proves that the IVMD algorithm proposed in this paper can effectively decompose signals. 

**Figure 12:**
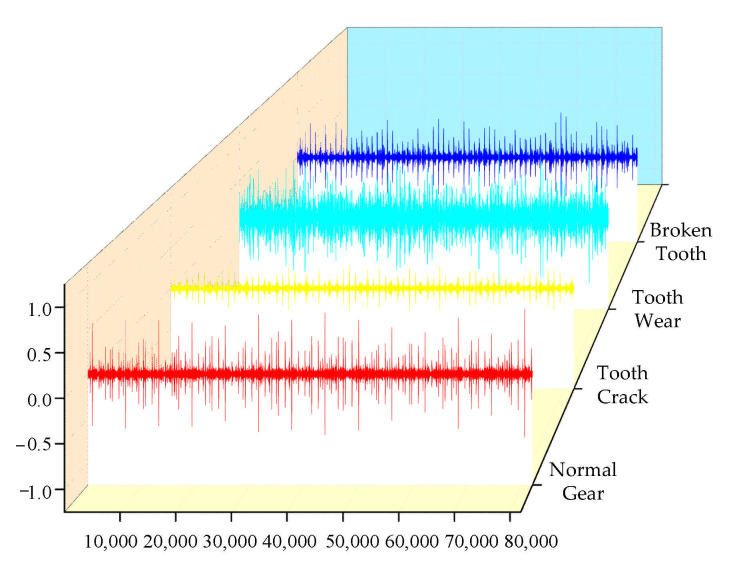
Time-domain waveform of the optimal IMFs.

In a gearbox system, a local fault in the gear causes periodic vibration of the signal. The more obvious the periodicity of the fault signal, the smaller the envelope entropy value. Therefore, the optimal mode components in each work stage were selected according to the minimum envelope entropy value criterion for subsequent feature vector extraction, and time-domain waveform of the optimal IMFs is depicted in Figure 12. 

### 6.2. Experimental Signal Feature Extraction Based on the TSMSE Algorithm

**Figure 13:**
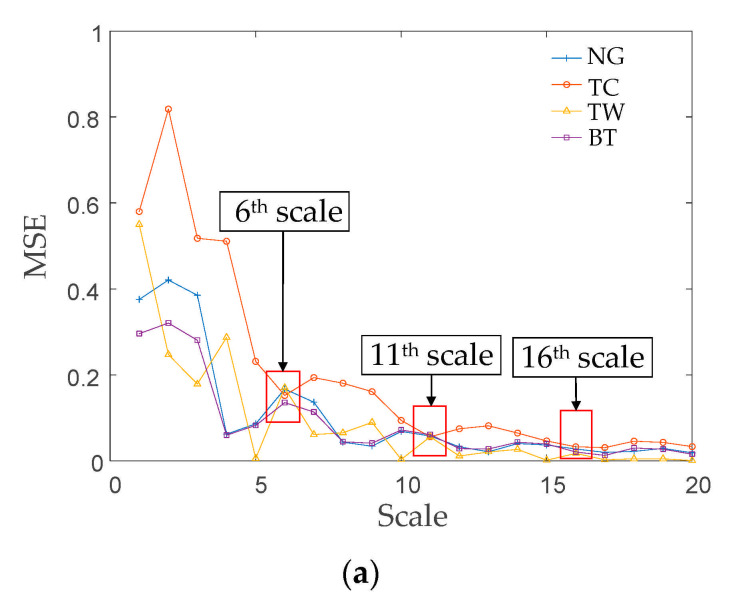

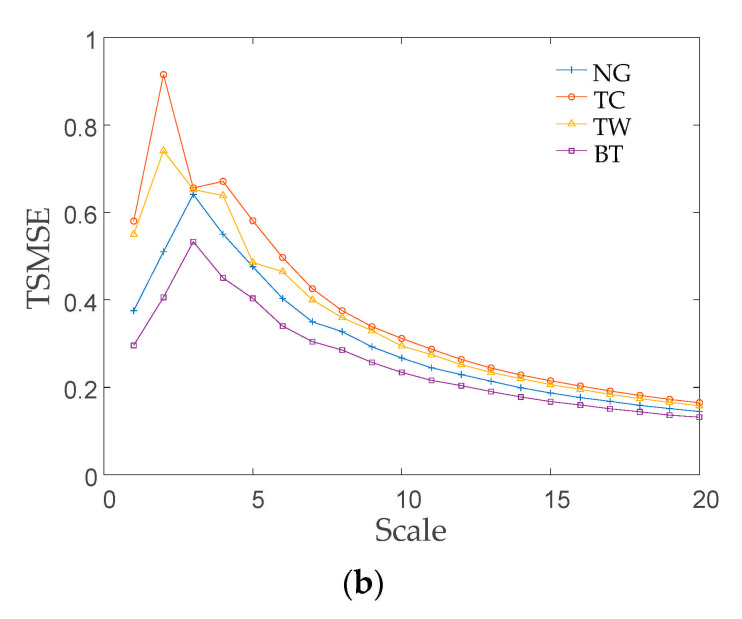
Feature entropy of signals of the four gearbox conditions. (**a**) MSE results of the signals of the four conditions; (**b**) TSMSE results of the signals of the four conditions.

In this experiment, the TSMSE algorithm was used to extract the features of the optimal mode components selected in Section 6.1. The TSMSE and MSE algorithms were separately applied to extract the features of the signals of the four conditions to prove that the proposed TSMSE can effectively distinguish the gear signals in various conditions. The results are shown in Figure 13, where, compared with the TSMSE algorithm, the MSE algorithm cannot effectively distinguish the different conditions of gear. In Figure 13a, the feature entropy extracted by MSE shows serious mixing. Specifically, the normal gear and broken tooth signals in the first four scales can be effectively distinguished. With the increase in the scale, the two entropy curves nearly overlap. Tooth crack and tooth wear signals only overlap on the 6^th^, 11^th^, and 16^th^ scale; on the other scales, the two signals can be identified. Considering the whole scale, the four features’ entropy curves seriously cross, which means the four signals’ conditions cannot be clearly distinguished. It also can be seen that MSE is unable to measure the complexity of the signal on higher scales due to the influence of coarse graining processing. The feature entropy obtained by the TSMSE algorithm shows a good degree of differentiation for each signal (Figure 13b), and there is almost no signal point crossing in the curves, which proves that the TSMSE algorithm can effectively distinguish gearbox signals under different conditions.

### 6.3. Experimental Signal Fault Classification Based on SSA-SVM Algorithm

Based on the above experimental signal analysis results, the SSA was applied to perform adaptive optimization of the parameters (*c*, *σ*) of the SVM. The parameters of the algorithm are shown in Table 1.

**Figure 14:**
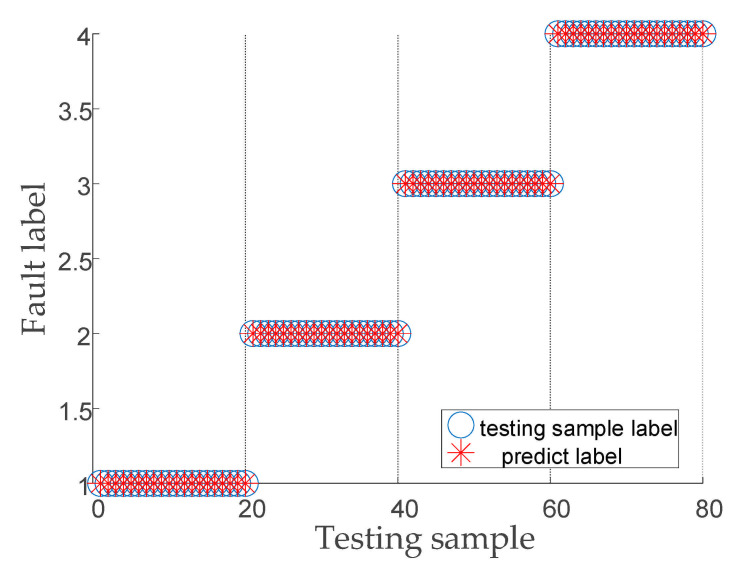
SSA-SVM classification results.

The feature entropy vectors from Section 6.1 were randomly and equally divided into a training sample and test sample, and the samples were used for training and testing of the SSA-SVM classification model. The optimal SVM parameters obtained from the search were [91.28, 1.78]. Figure 14 shows that the fault diagnosis model proposed in this paper has high identification accuracy (100%) and diagnosis capability of the vibration signals of wind turbine gearboxes.

The above analysis shows that the fault diagnosis model of mechanical equipment usually contains three parts, signal decomposition, fault feature extraction, and fault pattern identification. We optimized the above three parts separately and proved that the proposed optimization algorithm of each part (IVMD, TSMSE, SSA-SVM) is superior to the original algorithm (VMD, MSE, SVM) by comparative analysis. Furthermore, to prove that the final combination algorithm (IVMD-TSMSE-SSA-SVM) provides advantages in fault diagnosis of wind turbine gearbox, the proposed optimized combination algorithm model was compared with different combination algorithm models, and the results are shown in Table 2. The default VMD decomposition parameter of VMD was (3, 2000), and each method was repeated 5 times. From the table, three conclusions can be drawn:

(1) The average testing accuracy of the classification model using the IVMD algorithm (96.15%) is higher than that using the default VMD parameter (93.28%). Consequently, the above result proves that the proposed IVMD algorithm performs better than the default parameter VMD algorithm in signal decomposition.

(2) The average testing accuracy of 98.45% can be achieved by the classification model combined with the TSMSE feature vector, which outperforms the model with the feature vector selected by MSE (90.95%), as the TSMSE reflects the complexity and chaos of the signal on different scales and it is influenced by the length of the time series that is weakly compared with the MSE algorithm due to the time-shift technology. The above discussion verifies that the TSMSE algorithm can extract information with more abundant fault features and is superior in fault feature extraction.

(3) The SSA-SVM model has higher fault identification accuracy compared with the original SVM model; the testing accuracies of the two models are 97.05% and 92.35%, respectively, which indicate that the SSA can effectively determine the best SVM parameters. That is, the SSA-SVM model can effectively identify gear vibration signals under different conditions. 

The above analysis shows that the performance of the proposed model in wind turbine gearbox fault diagnosis is more accurate than that of other methods. Therefore, this model shows great application potential in fault diagnosis.

## 7. Conclusions

This paper proposed a new wind turbine gearbox fault diagnosis model to accurately extract fault features and diagnose fault states. The effectiveness of this model in fault diagnosis was verified through experimentation; however, the proposed model has some limitations, which are listed as follows:

(1) At present, the rotating machinery equipment is developing toward being large-scale and intelligent, so the structure of the equipment is becoming increasingly complex, and the collected signals contain increasingly abundant information, which poses difficulties in effectively extracting the fault features of a signal. Although the IVMD algorithm effectively determines the parameters of the VMD adaptively, the time cost increases when it deals with complex signals. Therefore, improving the IVMD algorithm to reduce the time cost is a direction for future research.

(2) In the proposed method, SSA is used to optimize the parameters of the SVM model. Although SSA has some advantages compared with other algorithms, the parameters (percentage of producers and safety threshold) in the SSA are set artificially, which relies on the users’ prior experience. This may lead to poor optimization results due to improper parameter selection. Therefore, future research should determine how to avoid the influence of human factors on the optimization effect of SSA.

## Figures and Tables

**Table 1:** SSA parameter settings.

Parameters	Setting
Population size	50
iter_max_	100
Percentage of producer	0.2
Range of parameter *c*	[1, 100]
Range of parameter *σ*	[1, 100]
ST	0.8

**Table 2:** Fault diagnosis accuracy of different combination algorithm models.

Signal Decomposition Algorithm	Fault Feature Extraction Algorithm	Classification Algorithm	Accuracy
IVMD	TSMSE	SSA-SVM	100%
IVMD	MSE	SSA-SVM	95.8%
VMD	TSMSE	SSA-SVM	98%
VMD	MSE	SSA-SVM	94.4%
IVMD	TSMSE	SVM	98.2%
IVMD	MSE	SVM	90.6%
VMD	TSMSE	SVM	97.6%
VMD	MSE	SVM	83.01%

## Data Availability

The data used in this study are all owned by the research groupand will not be transmitted.
